# MRI-Based Quantitative Assessment of Normal-Appearing White and Gray Matter Demyelination in Multiple Sclerosis, Parkinson’s Disease, Long COVID, and Normal Aging

**DOI:** 10.3390/biomedicines14081691

**Published:** 2026-07-28

**Authors:** Marina Khodanovich, Mikhail Svetlik, Daria Kamaeva, Anna Usova, Tatyana Anan’ina, Irina Wasserlauf, Maria Shadrina, Valentina Pashkevich, Marina Moshkina, Victoria Obukhovskaya, Nadezhda Kataeva, Anastasia Levina, Valentina Alifirova, Marina Titova, Irina Zhukova, Svetlana Vasilieva, Evgeny Schastnyy, Yana A. Tumentceva, Vasily Yarnykh

**Affiliations:** 1 Laboratory of Neurobiology, Research Institute of Biology and Biophysics, Tomsk State University, 36 Lenina Ave., Tomsk 634050, Russiairinawasserlauf@mail.ru (I.W.); shadrina@mail.tsu.ru (M.S.); nadi-51@yandex.ru (N.K.);; 2 Cancer Research Institute, Branch of the Tomsk National Research Medical Center of the Russian Academy of Sciences, Kooperativnyi per. 5, Tomsk 634009, Russia; 3 Department of Fundamental Psychology and Behavioral Medicine, Siberian State Medical University, 2 Moskovskiy Trakt, Tomsk 634050, Russia; 4 Department of Neurology and Neurosurgery, Siberian State Medical University, 2 Moskovskiy Trakt, Tomsk 634050, Russia; 5 Medica Diagnostic and Treatment Center, 86 Sovetskaya St., Tomsk 634034, Russia; 6 Department of Affective States, Mental Health Research Institute, Tomsk National Research Medical Center of the Russian Academy of Sciences, Tomsk 634014, Russia; vasilievasn@yandex.ru (S.V.);; 7 Department of Radiology, School of Medicine, South Lake Union Campus, University of Washington, 850 Republican Street, Seattle, WA 98109, USA; yarnykh@uw.edu

**Keywords:** myelin, quantitative MRI, neuroimaging, macromolecular fraction mapping, MPF, white matter, FLAIR hyperintensities, demyelination, multiple sclerosis, Parkinson’s disease, long COVID, depression, normal aging

## Abstract

**Background/Objectives**: Normal-appearing white (WM) and gray (GM) matter outside focal demyelinating lesions (WMH) has been found impaired in many diseases and normal aging. The present study aimed to compare global GM and WM alterations in patients with multiple sclerosis (MS), Parkinson’s disease, long COVID (LC) and normal aging. **Methods**: The study population comprised 196 participants including patients with MS (n = 42), PD (n = 16), LC (n = 75), and healthy volunteers (n = 63). All participants underwent MR scanning using the fast macromolecular fraction (MPF) mapping protocol and routine clinical sequences. MPF in global normal-appearing WM, GM, and mixed WM-GM was measured after exclusion of segmented focal lesions. To eliminate the influence of gender, age, and brain atrophy on the results, the corresponding covariates were included in the analysis. **Results**: Significant WMH volume increase and MPF decrease in global WM, WM-GM, and GM were found both in normal aging (75–85 years) and MS, PD, and LC patients (except for WM and WMH volume in LC patients). The greatest MPF decrease was observed in MS patients. The youngest (18–24 years) controls had a significant MPF decrease in global WM, WM-GM, and GM compared to middle-aged (35–44 years) participants. Among LC patients, the greatest global MPF reduction was found in patients with depression and insomnia as COVID-19 complications. In healthy controls, the peak age of myelination was estimated as 42.2 years for WM, 46.5 years for WM-GM, and 45.4 years for GM. Significant correlations were found between MPF and EDSS in MS patients (r = −0.71 in GM, r = −0.58 in WM-GM, r = −0.44 in WM) and stage of PD in PD patients (r = −0.58 in GM, r = −0.55 in WM-GM, r = −0.52 in WM). **Conclusions**: MPF mapping showed high sensitivity to age-related and disease-related differences in brain myelination, particularly in GM. Our results confirm the feasibility of using MPF mapping in large-scale clinical studies.

## 1. Introduction

Brain demyelination plays a key role in the pathology of primary demyelinating diseases, the most common of which is multiple sclerosis (MS). However, demyelination is often secondary to the underlying cause of the disease (infection, genetic factors, stress, etc.). This often leads to inflammation, ischemia, metabolic disturbances, and oxidative stress, with subsequent damage to neurons and oligodendrocytes, as well as myelin loss caused by pro-inflammatory cytokines, excitotoxicity, energy failure, dysregulation of myelin synthesis, and other molecular mechanisms [1,2,3]. The above pathological processes are observed in various neurological, psychiatric, and infectious conditions, including cerebral ischemia and stroke, postinfectious encephalitis, disseminated encephalomyelitis, Alzheimer’s disease, Parkinson’s disease (PD), schizophrenia, depression, and many others [4,5,6,7]. Post-mortem histopathology has also demonstrated a primary role of inflammation in the development of demyelination, particularly in MS [8,9]. Possible pathological mechanisms of demyelination after COVID-19 infection include inflammation, the direct effects of the virus on oligodendrocytes, and cerebrovascular complications of prolonged hypoxia [10,11,12,13]. Brain aging is also known to detrimentally affect myelin and white matter (WM) microstructure, predominantly through the pro-inflammatory pathway and oxidative stress [14,15] that is a common feature of many neuroinflammatory and neurodegenerative conditions including MS, Alzheimer’s disease, and PD [1,2,3,4,5,6,7].

The most remarkable and widely used diagnostic feature of demyelination is focal lesions, detected as areas of WM hyperintensity (WMH) on T2-weighted MR images [16,17,18]. Quantitative MRI methods are also capable of detecting microscopic myelin loss in normal-appearing WM (NAWM) and GM outside of focal lesions, which are invisible on traditional MR images. Longitudinal study by de Groot et al. [19] found that NAWM changes precede aging-related WMH on T2-weighted fluid-attenuated inversion recovery (T2/FLAIR) images. Decline in NAWM myelination has been found in MS [18,20,21,22,23], PD [5,7,24,25], schizophrenia [26], depression [27], and normal aging [28,29,30,31,32]. Furthermore, several studies provided evidence of GM demyelination in MS [21,23,33], schizophrenia [26], depression [27], and normal aging [31,32].

Among quantitative MRI methods, only a few have demonstrated sensitivity to myelin changes in GM [21,23,26,27,31,32,33]. One of them is macromolecular proton fraction (MPF) mapping [34], which measures the relative content of macromolecular protons causing the magnetization transfer (MT) effect in tissues [34,35]. This effect is manifested as magnetization exchange between protons of free water and macromolecules and is described theoretically by the two-pool equilibrium model [36] including MPF, cross-relaxation rate constant, and individual relaxation times of the proton pools as the parameters specifying magnetization dynamics. Despite the model complexity, MPF maps can be reconstructed in isolation from other two-pool model parameters using a recent single-point MPF mapping method [37,38], which requires only three source images and enables fast clinically feasible data acquisition.

Unlike the semi-quantitative index MT ratio (MTR), the MPF parameter showed independence on longitudinal relaxation [23] and paramagnetic effects caused by iron deposition [33]. Unlike widely used diffusion tensor imaging (DTI), MPF is independent of the direction of myelin fibers and can be correctly measured in regions containing multidirectional fibers [39]. According to MR-histology studies, MWF and MPF are generally considered more specific to myelin content, while DTI indices are more sensitive to WM microstructure and axonal integrity [40,41]. Numerous studies in animal models, including cuprizone-induced demyelination and remyelination [42,43], ischemic stroke [44,45], neonatal development [46], and normal animal brain [47], showed strong correlations between MPF and myelin histology. MPF mapping has demonstrated high reproducibility [48], independence on magnetic field strength [47], and easy implementation based on routine MRI equipment and original manufacturers’ pulse sequences [26,49]. Particularly, for commonly used clinical MRI platforms with 1.5 T and 3 T magnetic field strengths, the fast MPF method demonstrated highly consistent quantitative results [50] and robustness against variations in the signal-to-noise ratio [49]. Human studies have shown high sensitivity of MPF to myelin alterations in WM and GM in multiple sclerosis [23,33], mild traumatic brain injury [51], schizophrenia and psychotic spectrum disorders [26,52,53], post-COVID depression [27], normal aging [31,32], and brain development in fetuses [49], children [54], and adolescents [55].

Collectively, the above studies [23,26,27,31,32,33,49,51,54,55] suggest that MPF provides a uniform, highly reproducible quantitative myelin scale across a variety of neuropathological conditions and normal physiological states. Leveraging our large database including MPF maps from almost 200 participants obtained using the same MRI scanner and MPF protocol, this study sought to outline a broad landscape of myelin differences in several neurological diseases and normal aging. More specifically, the primary goal of this study was to evaluate disease-related global myelin differences in normal-appearing WM and GM in patients with MS, PD, and long COVID (LC) and compare them to healthy participants in separation from the influence of gender, age and brain atrophy. Additionally, we aimed to assess age-related myelin differences in a larger sample of healthy controls to refine the dependencies observed in our previous studies [31,32].

## 2. Materials and Methods

### 2.1. Participants

The study involved 196 participants in total. Patients with MS (n = 42), PD (n = 16), post-COVID complications (n = 75), and healthy volunteers (n = 63) were recruited from the Siberian State Medical University (Tomsk, Russia), the Mental Health Research Institute (Tomsk, Russia), the Medica Diagnostic and Treatment Center (Tomsk, Russia), and Tomsk State University. The common inclusion criterion for patients and controls was the age of 18 years or older. The common exclusion criteria for patients and controls were: a history of traumatic brain injury, acute infection or somatic diseases at the time of study, pregnancy, contraindications to MRI, inability to tolerate the MRI procedure, and self-withdrawal from the study. For the control group, an additional criterion was the absence of any diagnosed neurologic or psychiatric condition. For the MS and PD patients, additional inclusion criteria were the definite diagnosis of MS and PD, respectively. For the LC patients, additional inclusion criteria were: previously positive COVID-19 PCR test, persistence of post-COVID neuropsychiatric complications at the time of the study, and the absence of neurological or psychiatric diagnoses prior to COVID-19. LC patients were newly diagnosed by board-certified neurologists and psychiatrists, and the following symptom types were recorded: clinical depression, chronic headache, insomnia, asthenia, mild cognitive impairment, anosmia/hyposmia, ageusia, panic disorder, bipolar disorder, anxiety disorder, autonomic neuropathy, myalgia, dorsalgia, arthralgia, vestibular disorder, cervicobrachial syndrome, and disorder of the autonomic nervous system. Most of these complications are common for LC [12,13]. Subgroups were formed from the patients presenting with a particular symptom type if the number of such patients exceeded 10.

The demographic characteristics of participants are shown in Table 1.

All patients and healthy participants who met the inclusion and exclusion criteria signed an informed consent form. The study design was approved by the Bioethics Committee of the Cancer Research Institute (protocol No. 3 dated 26 May 2017), the Ethical Committee of Siberian State Medical University (protocol No. 5652 dated 27 November 2017), the Ethical Committee of the Mental Health Research Institute (protocol No. 15 dated 25 August 2022) and Bioethics Committee of Tomsk State University (No. 12 dated 6 June 2022) following the guidelines of the Declaration of Helsinki.

Considering the previously revealed significant influence of gender on MPF and the quadratic association of MPF on age in healthy individuals [31,32], the differences between patient groups and the control group by gender, mean age, and age range were analyzed (Table 1, Supplementary Table S1, Supplementary Figure S1). Since all patient groups differed significantly from the control group in mean age (PD patients), gender (MS patients), and age range (MS, PD, and LC patients), age- and gender-related covariates were used for correct intergroup comparison.

To study the dependence of MPF and WMHs on age, all healthy study participants were divided into the following age groups: 18–24 (n = 14), 25–34 (n = 12), 35–44 (n = 10), 45–54 (n = 11), 55–64 (n = 6), 65–74 (n = 6), and 75–85 (n = 4) years old.

### 2.2. MRI Data Acquisition

All participants underwent MR imaging using a 1.5 T clinical scanner, Magnetom Essenza (Siemens, Erlangen, Germany), with an 8-channel head coil. The standard MRI protocol included:

- 3D T2-FLAIR: TR = 5000 ms, TE = 390 ms, TI = 1800 ms;
- 3D T1-weighted: TR = 16 ms, TE = 4.76 ms;
- 3D T2-weighted: TR = 3000 ms, TE = 335 ms;

The fast MPF mapping protocol [26] included three 3D spoiled gradient-echo pulse sequences with the following parameters:

- MT-weighted: TR = 20 ms, echo time (TE) = 4.76 ms, flip angle (FA) = 8°, scan time 5 min 40 s;
- T1-weighted: TR = 16 ms, TE = 4.76 ms, FA = 18°, scan time 4 min 32 s;
- Proton-density-weighted: TR = 16 ms, TE = 4.76 ms, FA = 3°, scan time 4 min 32 s.

All scans were acquired in the sagittal plane with a voxel size of 1.25 × 1.25 × 1.25 mm^3^ (matrix 192 × 192 × 160, field of view 240 × 240 × 200 mm^3^), and single signal averaging. In the MT-weighted sequence, a standard manufacturer’s off-resonance saturation pulse was used. This pulse has a Gaussian envelope, offset frequency of 1.5 kHz, effective FA of 500°, and duration of 7.68 ms. Scan-rescan repeatability of its protocol and automated whole-brain tissue segmentation was assessed earlier [26].

The total scanning time was about 35 min.

### 2.3. Image Processing

MPF maps were reconstructed from T1w, MTw, and PDw images using a single-point algorithm with a synthetic reference image [38], which was implemented by the previously developed software in the C++ language (version 2.0, available at https://www.macromolecularmri.org).

**Figure 1:**
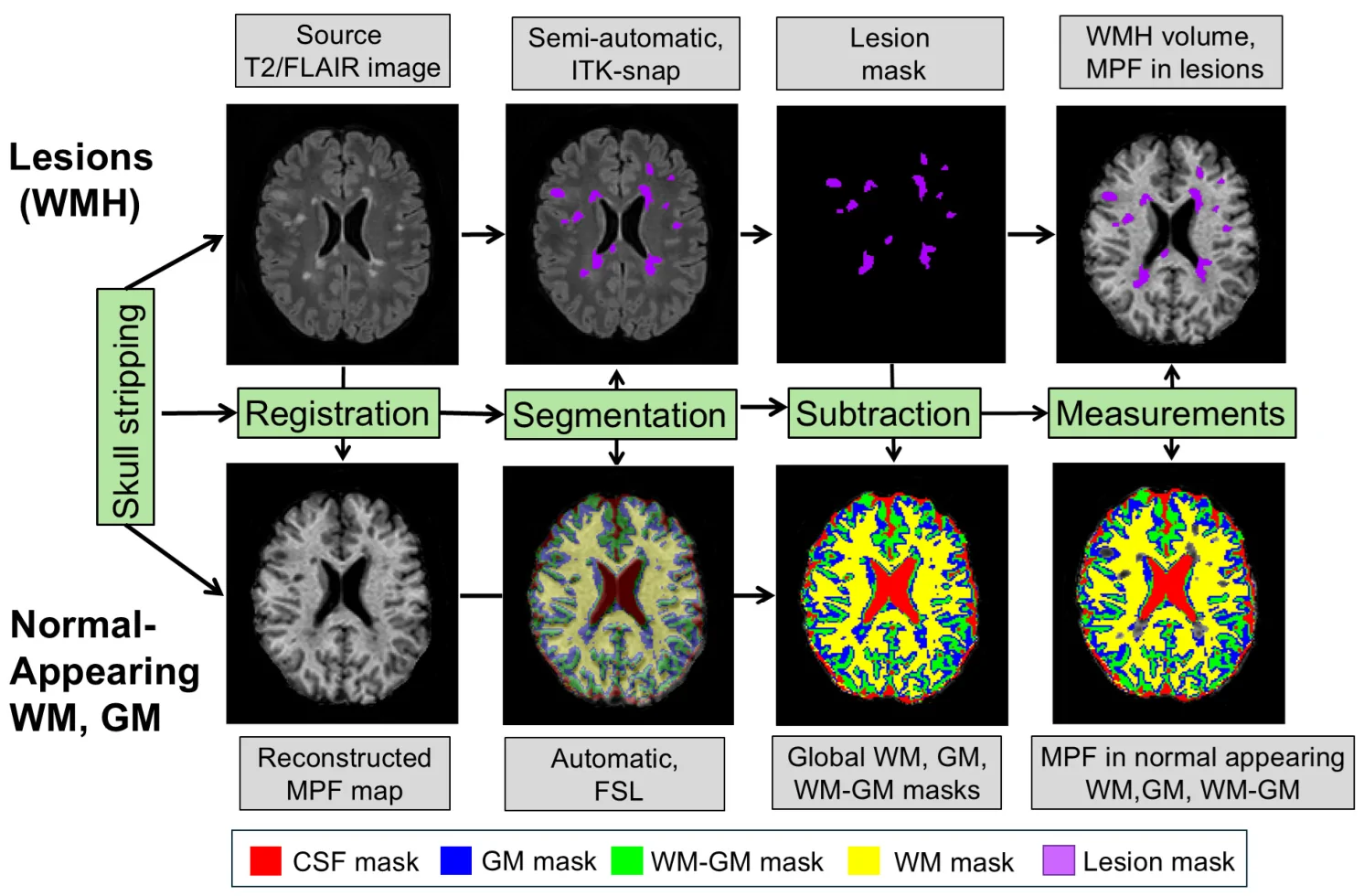
Scheme of the image processing pipeline (in the figure: patient with MS, female, 35 years old): skull stripping, registration, segmentation, subtraction, and measurements. The skull-stripped source T2/FLAIR image was registered to an MPF map reconstructed from T1w, MTw, and PDw images, used for semi-automatic lesion segmentation, obtaining a lesion mask, and measuring WMH volume and MPF inside lesions. Automatic FSL segmentation was performed using MPF maps to obtain WM, WM-GM, and GM global masks. A lesion mask was subtracted from the WM, WM-GM, and GM global masks, and the subtraction result was used to measure MPF in global compartments.

Image processing included the following steps (Figure 1):

(1) Skull stripping was performed using a mask, which was obtained by applying the BET algorithm to the PD-weighted images in the MRIcro application (Version 1.40) [56]. The mask was converted to a binary image and applied to the MPF maps to remove extracerebral tissue.
(2) WMH were segmented using the semi-automatic active contour (“snake”) tool of the ITK-snap application (version 3.6.0) by one operator blinded to the subject information on T2/FLAIR images with the guidance of T2-weighted and T1-weighted images.
(3) T2/FLAIR images were registered to MPF maps using ITK-snap software to measure WMH volumes and mean MPF values in the outlined areas.
(4) Automatic global segmentation of MPF maps was performed using the FSL package (version 6.0.1) [57] to obtain masks of WM, GM, mixed WM-GM, and mixed GM with cerebrospinal fluid (CSF) compartments as detailed earlier [23,26]. The segmentation procedure was initialized with the tissue priors of 13%, 6%, 9%, and 1% for WM, GM, WM-GM, and CSF, respectively. The four-class segmentation was used to separate MPF measurements in pure WM and GM from the effect of partial volume averaging in mixed (WM-GM and GM-CSF) tissue classes. The mixed WM-GM compartment mainly included voxels at the cortical GM-WM boundary and subcortical GM structures (basal ganglia, thalamus), in which GM was interspersed with WM tracts.
(5) To obtain MPF measurements of normal-appearing white and gray matter outside of focal lesions, WMH masks were subtracted from global compartment masks. The resulting masks were used to measure average MPF values for the global compartments. The measurements were carried out using the ITK-SNAP application [58].

### 2.4. Statistical Analysis

Statistical analysis was performed using Statistica 10.0 software. The following tests were used to assess differences between groups in demographic characteristics: Fisher exact test for gender ratio, Fisher LSD test for mean age, and Levene’s test of homogeneity of variance for age range. The results of Levene’s test of homogeneity of variance were presented as raincloud plots using JASP software (version 0.96). Initially (Preliminary Model in Supplementary Material, Text S1), all patient groups were compared with the control group using one-way ANOVA followed by post hoc Bonferroni and Welch tests.

To construct a more accurate model that takes into account the significance of differences in age between groups, a regression analysis of the dependence of MPF on age was carried out. The relationship between age and MPF in global compartments was investigated for the total sample of healthy participants and separately for men and women by fitting a quadratic function. The significance of the quadratic function was accepted if all three coefficients of the quadratic equation were significant according to t-values and the overall model was significant according to the F-test (Likelihood Ratio test). The peak age of myelination was calculated from the coefficients of quadratic equations for the total sample of each group and for men and women separately. The significance of the differences between the models for male and female healthy participants was assessed by the overlap of the confidence intervals of the coefficients of equations and using the Chow Test (F-Test for Global Difference). A leave-one-out analysis was performed for each model. The selection of observations for exclusion was based on the largest absolute value of standardized residual within the resulting model. The residuals obtained after fitting the model and excluding the extreme point were tested for normality using the Shapiro–Wilk test (Supplementary Material, Table S6). The obtained quadratic equations of the relationship between MPF and age, unique to each compartment (f(age): MPF_WM_ = 12.62 + 0.069 × age − 0.0008 × age^2^, MPF_WM-GM_ = 8.94 + 0.077 × age − 0.0008 × age^2^, and MPF_GM_ = 6.50 + 0.057 × age − 0.00065 × age^2^, Supplementary Material, Table S5) were used to transform the age variable for main analyses. The resulting variables (f(age)) were used as covariates in the subsequent main analysis of differences between groups to eliminate the influence of age on the results.

Main analysis (Main Model in Supplementary Material Test S1) was performed using a General Linear Model (GLM) framework applied separately to each quantitative parameter (WMH and MPF in WM, WM-GM, and GM). This model was used to evaluate main clinical cohorts (PD, MS, and LC patients vs. controls), MS subtypes (RRMS and SPMS vs. controls), and individual LC symptom profiles (symptom-specific LC patients vs. controls and LC patients without the corresponding symptom). For all parameters, the models evaluated the main effects of patient group, sex, their interaction (Group × Sex), compartment volume (adjusting for brain atrophy), and parameter-specific age effects (linear “age” covariate for WMH and tissue-specific quadratic f(age) for MPF). To control for a possible effect of brain atrophy, age- and sex-dependence, three covariates were included in all analyses: (1) gender, (2) f(age), (3) the volumes of WM, WM-GM, and GM compartments in the respective tissue classes. To meet GLM assumptions, WMH data were rank-transformed (Conover–Iman procedure) prior to analysis to correct for high age-dependent variance and restore residual normality. Pairwise intergroup differences were evaluated using the post hoc Bonferroni test. The complete mathematical equations and model specifications are available in Supplementary Material (Text S1). All analyses were conducted according to [59].

Additionally, clinical significance of MPF measurements (raw and adjusted MPF) was analyzed using clinical data specific for each patient group. For MS patients, relationships between MPF and clinical data (Expanded Disability Status Scale (EDSS), disease duration) were assessed using Pearson’s r correlation coefficient. For PD and LC patients, associations between MPF and disease duration were assessed using Pearson’s r correlation coefficient and associations between MPF and disease severity were assessed using Spearman rank correlation. For PD patients, the severity of PD was assessed according to the Hoehn and Yahr scale (1–4). For LC patients, the severity of the acute phase of COVID-19 (1—mild, 2—moderate, 3—severe, 4—critical) was used. Additionally, to assess the influence of symptom overlap on MPF within the LC cohort, the Specific Model of GLM was used (Specific Model in Supplementary Material, Text S1). In particular, to investigate the overlap between the most frequently occurring symptoms of insomnia and depression, subgroups with isolated insomnia, isolated depression, comorbid insomnia and depression, LC patients without these symptoms, and healthy controls were identified. The model further controlled for acute disease severity and time since COVID-19 recovery along with sex, age, and brain atrophy. Medication status was omitted as all participants were treatment-naive at enrollment. Pairwise differences were also evaluated using the post hoc Bonferroni test.

Statistical significance for all analyses was considered as less than 0.05.

## 3. Results

### 3.1. Initial Comparison of Patient Groups with the Control Group Without Taking into Account the Influence of Gender, Age and Brain Atrophy

Table 2 and Supplementary Table S2 show the differences in MPF and WMH volume between patients and the control group. MS and PD patients demonstrated significant decreases in MPF across all global compartments and significant increases in WMH volumes. In LC patients, a significant decrease in MPF was found only in the WM-GM compartment.

### 3.2. Age-Related Global Differences in the Brain Myelination

**Figure 2:**
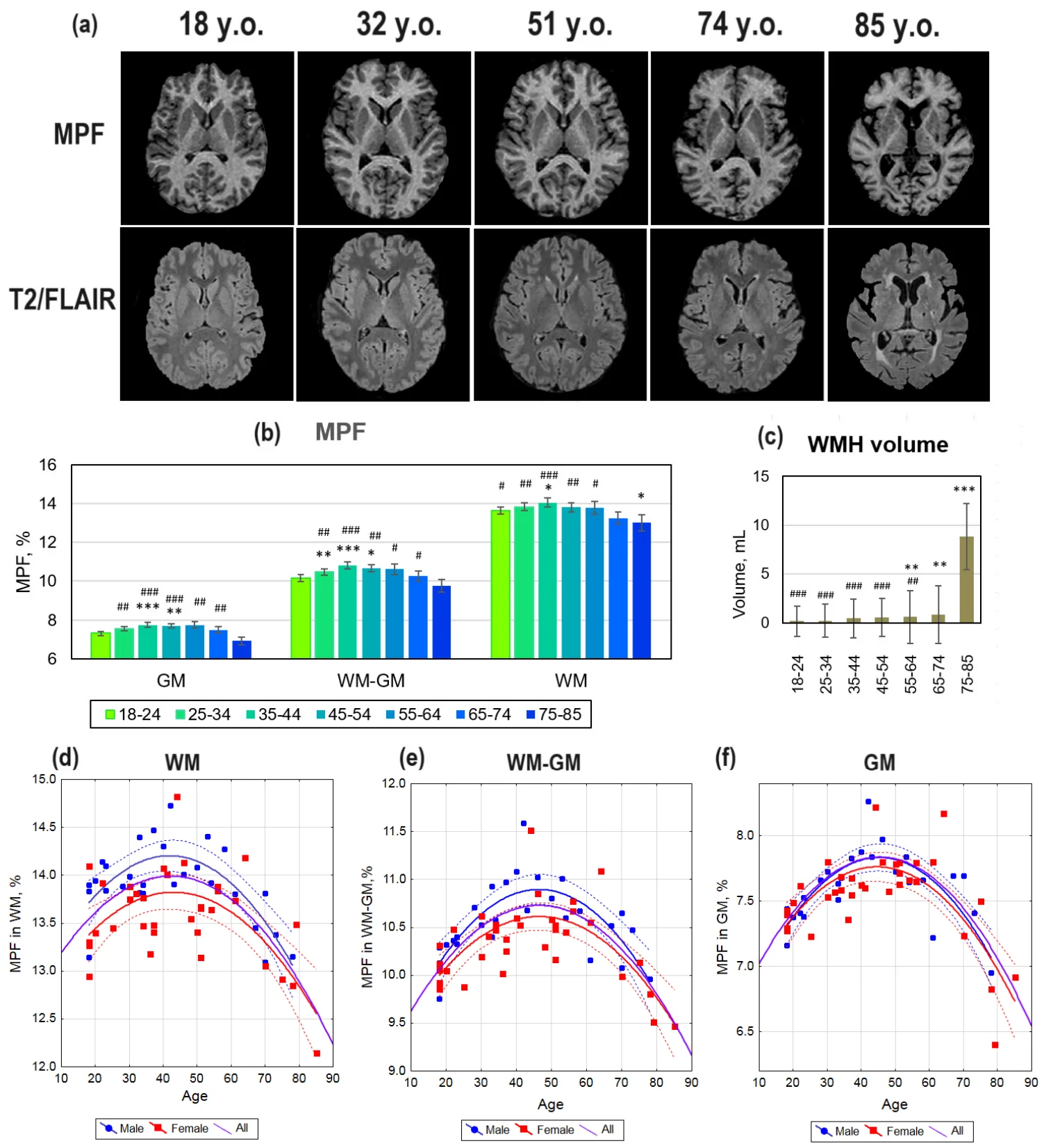
Age-related global differences in brain myelination. (**a**) Example MPF maps and T2/FLAIR images of healthy participants of different age groups: 18 years (female), 32 years (female), 51 years (female), 74 years (female). (**b**) Age-related MPF differences in GM, WM, and WM-GM global compartments. (**c**) Age-related differences in WMH volumes. (**d**–**f**) Quadratic model of MPF as a function of age for WM (**d**), WM-GM (**e**), and GM (**f**) obtained for men (blue line), women (red line), and the total sample (purple line). For quadratic models (**d**–**f**), the confidence intervals are shown with red and blue dotted lines for women and men, respectively. Error bars denote confidence intervals. Histograms (**b**,**c**) present MPF means, adjusted for gender and brain atrophy; scatterplots (**d**–**f**) show raw MPF values. Significant differences relative to the youngest (18–24 years) participants: *—*p* < 0.05, **—*p* < 0.01, ***—*p* < 0.001. Significant differences relative to the oldest (75–85 years) participants: #—*p* < 0.05, ##—*p* < 0.01, ###—*p* < 0.001.

Figure 2a shows examples of MPF maps and T2/FLAIR images of healthy study participants across different age ranges. In participants aged 18 and 32 years, no visible WMHs were detected on T2/FLAIR images. Participants aged 51 and 74 years showed typical age-related small WMHs, located predominantly periventricularly. In the 85-year-old participant, the WMH area in the cross-section on the T2/FLAIR image occupied a larger area compared to the 51- and 74-year-old participants. Quantitative assessment showed a significant increase in WMH volumes only for the 75–85-year age range compared to the 18–64 age range (Figure 2c).

No visible differences were observed between MPF maps of 18-, 32-, 51-, and 74-year-old participants. The MPF map of the 85-year-old participant had a prominent decrease in the contrast between WM and GM. Quantitative assessment of age-related differences (Figure 2b; Supplementary Tables S3 and S4), adjusted for gender and compartment volume, showed the highest MPF values in participants in the age range of 35–44 years, and the lowest MPF values in participants aged 75–85 years, for all compartments. MPF values in participants aged 75–85 years were significantly lower than those in participants aged 18–64 years in the WM compartment, lower than those in participants aged 25–74 years in the WM-GM compartment, and lower than those in participants aged 25–74 years in the GM compartment. Significantly lower MPF values relative to the average age range were also found for the youngest participants. Participants aged 18–24 years showed significantly lower MPF than those in participants aged 25–64 years in the GM and in participants aged 35–64 years in the WM-GM compartment. In WM, MPF in participants aged 18–24 years was significantly lower than that in participants aged 35–44 years but higher than that in participants aged 75–85 years. The effect of the tissue volume was significant for the WM and GM compartments (Supplementary Material, Tables S3 and S4).

Figure 2d–f demonstrates the results of fitting quadratic curves to the dependence of MPF on age, for the total sample of healthy controls and for men and women separately. The resulting equations for the total control sample were: MPF_WM_ = 12.62 + 0.069 × age − 0.0008 × age^2^, MPF_WM-GM_ = 8.94 + 0.077 × age − 0.0008 × age^2^, and MPF_GM_ = 6.50 + 0.057 × age − 0.00065 × age^2^. For all quadratic models, all three coefficients of the regression equation were significant (*p* < 0.001). Residuals after model fit did not show deviation from the normal distribution. Quadratic curves for women had a flatter shape than for men, which corresponds to a lower MPF peak in women in comparison with men. The peak age calculated from the regression equations for the total sample was 42.2 years, 46.6 years, and 45.4 years for the GM, WM-GM, and WM compartments, respectively. In men, the vertex of the quadratic curve was shifted relative to women towards younger ages for WM (41.7 years vs. 43.1 years), whereas the peak positions for WM-GM and GM were rather similar in both genders (Figure 2). All parameters of the quadratic models of the dependence of MPF on age are presented in Supplementary Table S5. The confidence intervals of the coefficients of equations for men and women overlapped. No significant differences in regression models for men and women were found according to the Chow Test (F-Test for Global Difference). The results of a Leave-One-Out analysis showed that all models remained significant after the exclusion of a single extreme data point; the coefficients of the quadratic equations differed by no more than 15%, and the residual distributions for both models (full sample and sample with the extreme data point excluded) did not differ from a normal distribution (Supplementary Table S6).

### 3.3. Diffuse and Focal Demyelination in MS Patients, Adjusting for Age, Gender, and Brain Atrophy

**Figure 3:**
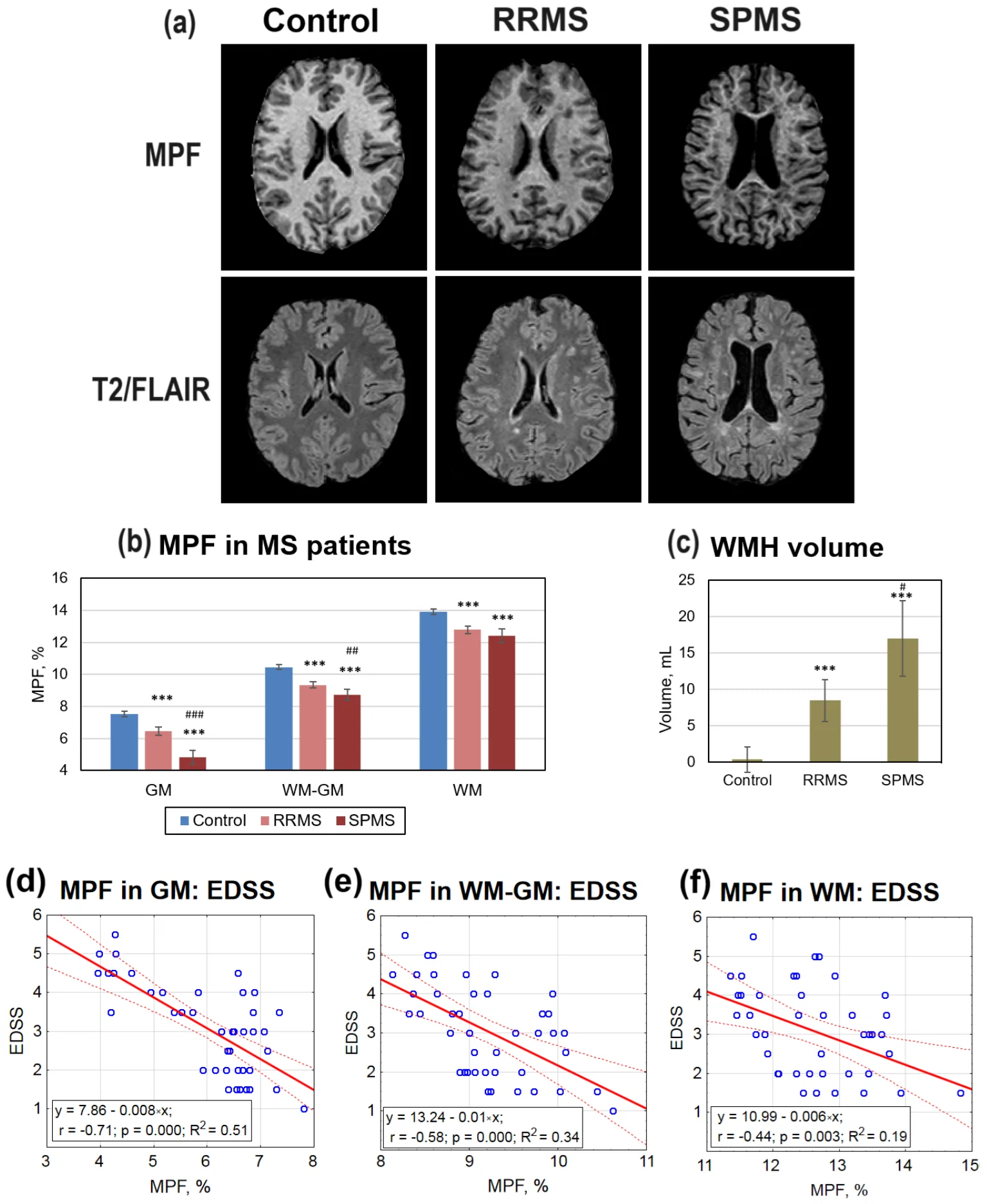
Global and focal changes of brain myelination in MS patients. (**a**) Example MPF maps of a healthy participant (female, 32 years), RRMS (female, 32 years), and SPMS (female, 32 years) patients. (**b**) MPF decrease, adjusted by age, gender, and brain atrophy, in global GM, WM, and WM-GM compartments in RRMS and SPMS patients relative to the control group. (**c**) WMH volume changes in RRMS and SPMS patients relative to controls. Error bars denote confidence intervals. Histograms (**b**,**c**) present MPF means (**b**), adjusted for age, gender, brain atrophy, and raw WMH values (**c**). Scatterplots (**d**–**f**) show raw MPF. Significant differences relative to the control group: ***—*p* < 0.001. Significant differences between RRMS and SPMS patients: #—*p* < 0.05, ##—*p* < 0.01, ###—*p* < 0.001. (**d**–**f**) Linear regressions of MPF in GM (**d**), WM-GM (**e**), and WM (**f**) on EDSS in MS patients. Solid red lines depict the plots of the regression equations. The 95% confidence intervals are shown as dotted red lines.

Figure 3 shows differences in brain myelination, adjusted by age, gender, and brain atrophy, between MS patients and the control group. Patients with MS showed a typical pattern of multiple focal lesions (WMH) on T2/FLAIR images, more prominent in SPMS patients (Figure 3a). These WMHs are clearly visible as hypointensities on the MPF maps (Figure 3a). SPMS patients also exhibited ventricular enlargement and lower WM/GM contrast on MPF maps. WMH volume was significantly higher in both RRMS and SPMS patients compared to the control group (Figure 3c; Supplementary Table S3). In GM and WM-GM compartments, MPF values, adjusted for age, gender, and compartment volume, were significantly lower in both RRMS and SPMS patients compared to the control group, and in SPMS patients compared to RRMS patients (Figure 3b; Supplementary Table S3). In normal-appearing WM, MPF values were significantly lower in both RRMS and SPMS patients compared to the control group, but no significant difference was found between RRMS and SPMS patients. The effect of age was significant in all MPF-related comparisons (Supplementary Table S4). The effects of gender and tissue volume were significant for the WM-GM compartment (Supplementary Table S4). A significant strong Pearson’s correlation was found between EDSS and MPF in GM (r = −0.71, *p* < 0.001; Figure 3d). Significant moderate correlations were found between EDSS and MPF in WM-GM (r = −0.58, *p* < 0.001; Figure 3e) and WM (r = −0.44, *p* < 0.01; Figure 3f). Weaker but significant correlations were found between MPF and MS duration (r = −0.32 for WM, *p* < 0.05; r = −0.31 for WM-GM, *p* < 0.05; r = −0.49 for GM, *p* < 0.001). However, Pearson’s correlations between adjusted MPF and EDSS were non-significant for all compartments (*p* > 0.05).

### 3.4. Diffuse and Focal Demyelination in PD Patients, Adjusting for Age, Gender, and Brain Atrophy

**Figure 4:**
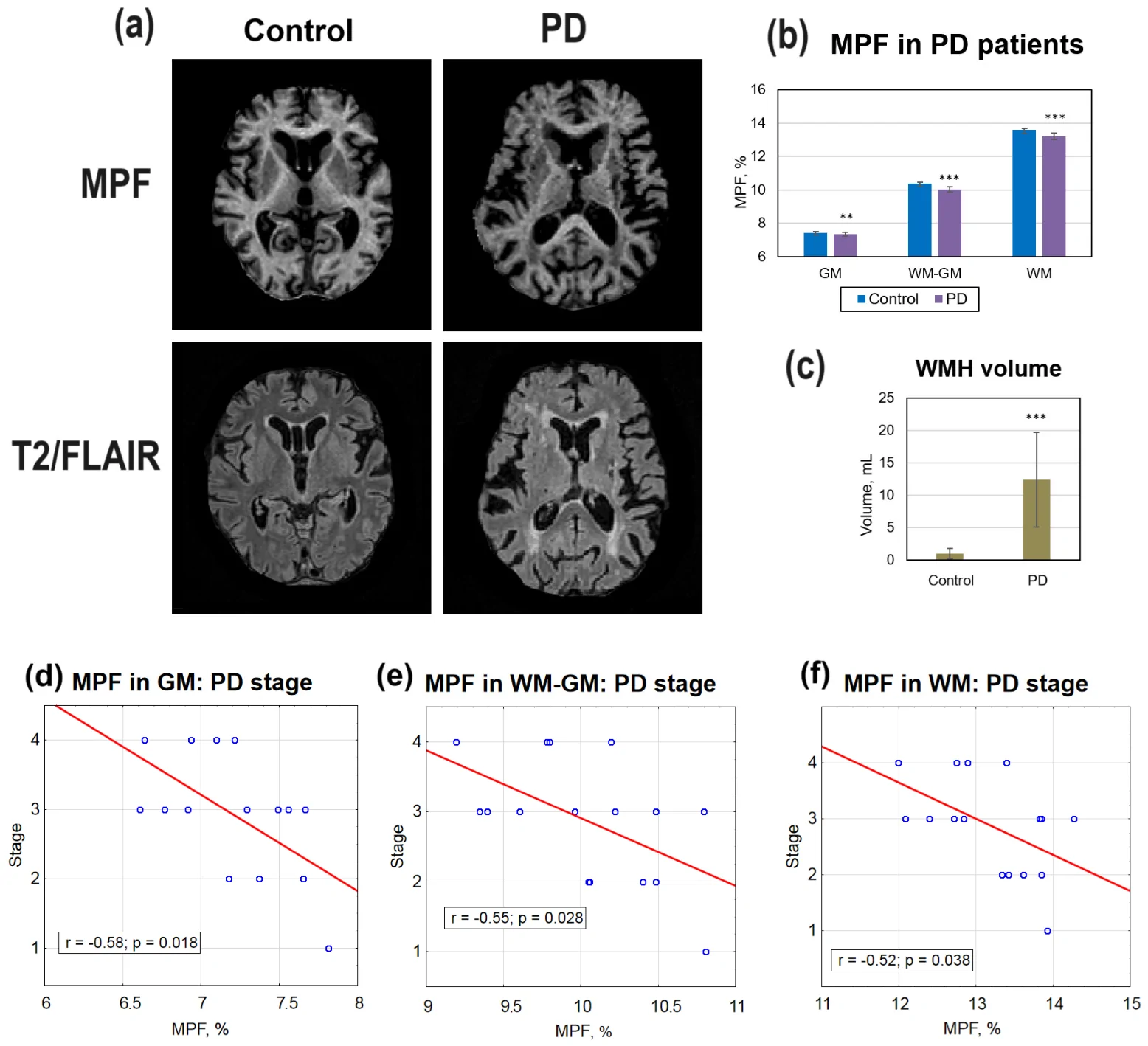
Changes of brain myelination in PD patients: (**a**) Example MPF maps of a healthy participant (male, 73 years) and a PD patient (male, 70 years). (**b**) MPF decrease in global GM, WM, and WM-GM compartments, adjusted by age, gender, and brain atrophy, in PD patients relative to the control group. (**c**) WMH volume differences in PD patients relative to controls. Error bars denote confidence intervals. Histograms (**b**,**c**) present MPF means (**b**), adjusted for age, gender, brain atrophy, and raw WMH values (**c**). Scatterplots (**d**–**f**) show raw MPF values. Significant relative to the control group: **—*p* < 0.01, ***—*p* < 0.001. (**d**–**f**) Relationships between MPF in GM (**d**), WM-GM (**e**), and WM (**f**) and stage of PD according to the Hoehn and Yahr scale. Solid red lines show the linear trends of relationships. Spearman correlation coefficients and their significance are shown on each scatterplot.

Differences in brain myelination, adjusted by age, gender, and brain atrophy, in patients with PD compared to the control group are shown in Figure 4. The area of WMH on T2/FLAIR images was larger in PD patients in comparison with controls (Figure 4a). Images of a PD patient also showed a visible pattern of brain atrophy and lower GM/WM contrast on the MPF maps (Figure 4a). The average WMH volume in PD patients was significantly higher compared to the control group (Figure 4c; Supplementary Table S3). MPF values, adjusted for age, gender, and compartment volume, in all compartments were lower in PD patients compared to controls, but these differences were significant only for GM and WM-GM (Figure 4b; Supplementary Table S3). The effect of age was significant for all MPF and WMH comparisons (Supplementary Table S4). The effect of tissue volume was significant for the WM-GM compartment (Supplementary Table S4). Significant moderate Spearman correlations were found between stage of PD (according to the Hoehn and Yahr scale) and MPF in GM (r = −0.58, *p* < 0.05; Figure 4d), WM-GM (r = −0.55, *p* < 0.05; Figure 4e), and WM (r = −0.52, *p* < 0.05; Figure 4f). The significance of the Spearman correlations between adjusted MPF and PD stage holds for the WM-GM (r = −0.50, *p* < 0.05) and GM (r = −0.61, *p* < 0.05) compartments and is close to significant for the WM (r = −0.43, *p* = 0.09).

### 3.5. Demyelination in LC Patients, Adjusting for Age, Gender, and Brain Atrophy

**Figure 5:**
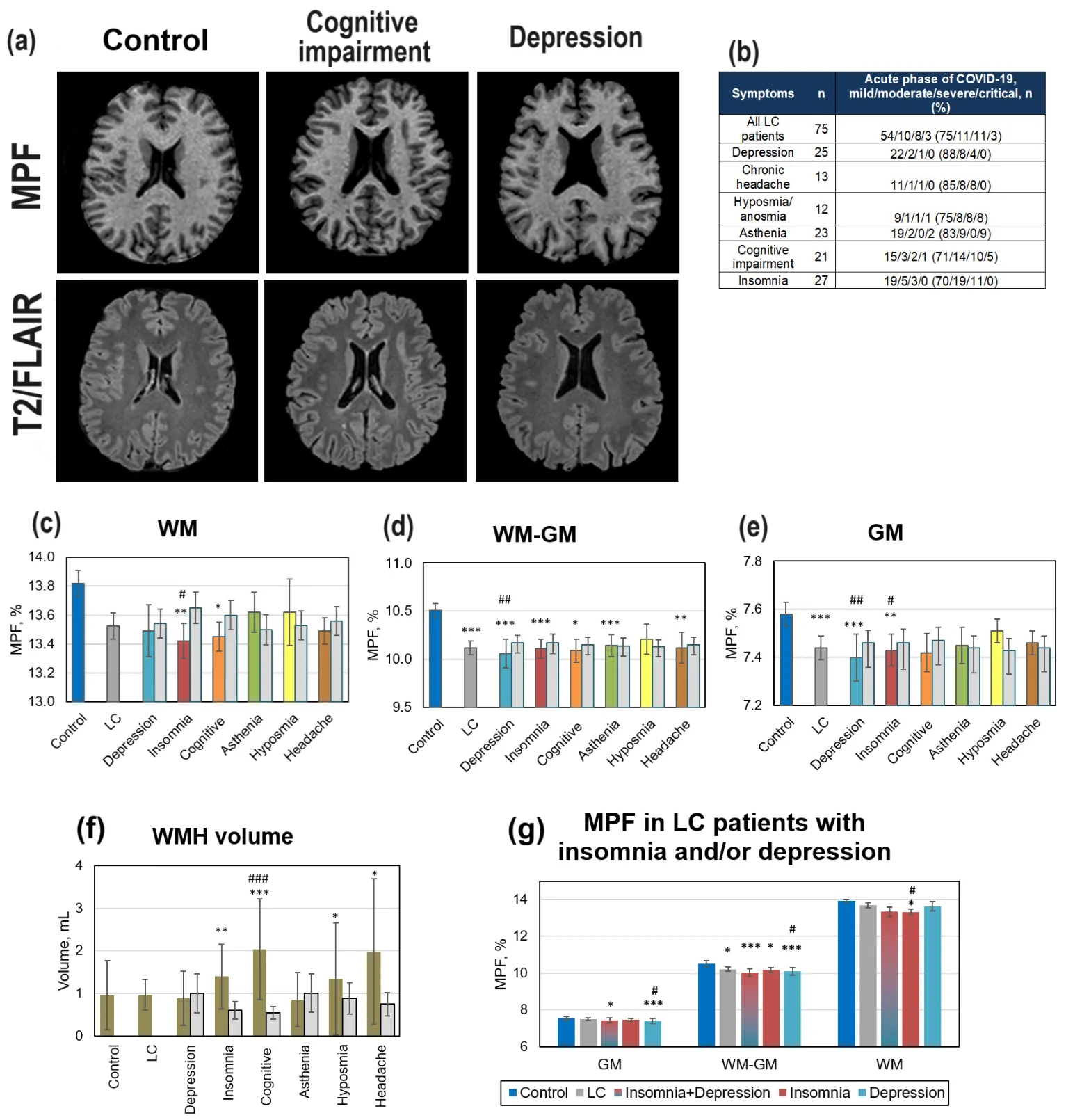
Global differences of brain myelination in LC patients: (**a**) Example MPF maps of a healthy participant (male, 46 years), LC patient with cognitive impairment (male, 48 years), and LC patient with depression (male, 46 years). (**b**) Number and COVID-19 severity of examined LC patients with clinically diagnosed specific LC symptoms of depression, chronic headache, hyposmia/anosmia, mild cognitive impairment, and insomnia. (**c**–**e**) MPF differences in global GM, WM, and WM-GM compartments, adjusted by age, gender, and brain atrophy, in LC patients with specific symptoms relative to the control group and LC patients without specific symptoms. (**f**) WMH volume in LC patients with specific symptoms. (**g**) MPF in LC patients with isolated and overlapped symptoms of insomnia and depression, adjusted for age, sex, brain atrophy, severity and time of recovery after the acute COVID-19. Light gray bars near each bar for LC patients with a specific symptom correspond to LC patients without this symptom/Error bars denote confidence intervals. Histograms (**c**–**e**) present MPF means, adjusted for age, gender, and brain atrophy. Histogram (**g**) shows MPF means, adjusted for age, gender, brain atrophy, COVID-19 severity, and time since recovery; Histogram (**f**) shows raw WMH values. Significant differences relative to the control group: *—*p* < 0.05, **—*p* < 0.01, ***—*p* < 0.001. Significant differences relative to other LC patients: #—*p* < 0.05, ##—*p* < 0.01, ###—*p* < 0.001.

Differences in brain myelination in LC patients (Figure 5) adjusted by age, gender, and brain atrophy relative to the control group were analyzed for both the total sample of LC patients and the LC patients with specific neuropsychiatric symptoms clinically diagnosed by neurologists or psychiatrists. The number of LC patients with specific symptoms (depression, chronic headache, hyposmia/anosmia, asthenia, mild cognitive impairment, insomnia) is shown in Figure 5b. Small WMHs are visible on the T2/FLAIR image of an LC patient with mild cognitive impairment after COVID-19 (Figure 5a). No visible differences on the MPF map in an LC patient with depression compared to a healthy participant are observed (Figure 5a).

Within the Main Model, WMH volume was significantly increased relative to controls in the LC patients with insomnia, headache, and mild cognitive impairment (Figure 5f). However, a significant reduction in WMH volume compared to other LC patients was observed only in LC patients with mild cognitive impairment. More pronounced differences were observed in MPF, adjusted for age, sex, and tissue volume: a significant MPF reduction compared to controls was observed in all compartments in the total sample of LC patients (Figure 5c–e; Supplementary Table S3). In the WM-GM (Figure 5c,d). significant MPF decrease was found for all subsamples corresponding to specific clinically diagnosed LC complications (except for hyposmia). MPF in WM showed a significant decrease for the LC patients with insomnia and cognitive impairments. In the GM, a significant MPF decrease compared to controls was found in the LC patients with depression and insomnia. Only in LC patients with insomnia and/or depression was a significant MPF reduction compared to other LC patients found. The effect of age was significant for all MPF and WMH comparisons (Supplementary Table S4). The effect of GM volume was significant for all subgroups of LC patients, except for depressed LC patients and patients with hyposmia, asthenia, insomnia, and cognitive impairments, for the WM compartment (Supplementary Table S4). The significant effect of gender was found for WM-GM (all comparisons) and WM (all comparisons except for the LC patients with depression and/or headache). Spearman rank correlations between MPF and the severity of the acute phase of COVID-19 and disease duration were not significant (*p* > 0.05).

The clinically diagnosed symptoms of LC overlapped significantly. Two of the six investigated clinical features were diagnosed in 30 patients (40.0%), three in 6 patients (8.0%), four in 6 patients (8.0%), and five in 1 patient (1.3%). Insomnia most frequently combined with depression (13 patients, 17.3%), cognitive impairment (13 patients, 17.3%), and asthenia (9 patients, 12.0%). Other combinations were observed in 6 patients (8.0%) or fewer. Additional statistical analyses within the Specific Model (Figure 5g; Supplementary Material, Text S1) showed that compared to controls, the MPF reduction remained significant for all isolated and combined neuropsychiatric phenotypes in the WM-GM compartment. In contrast, in the GM, this reduction was significant only for the isolated depression and combined insomnia-depression subgroups, whereas in the WM, it was restricted to LC patients with isolated insomnia (Figure 5g). Furthermore, the direct comparison with other LC patients showed that individuals with isolated depression exhibited significantly decreased MPF in both the GM and WM-GM compartments, while patients with isolated insomnia showed decreased MPF specifically within the WM. The factors “disease severity” and “time since COVID-19” were found to be non-significant; only the subgroup factor and the age-related quadratic covariate reached statistical significance.

### 3.6. Comparison of Global Demyelination and WMH Volume in MS, PD, LC Patients, and Normal Aging

**Figure 6:**
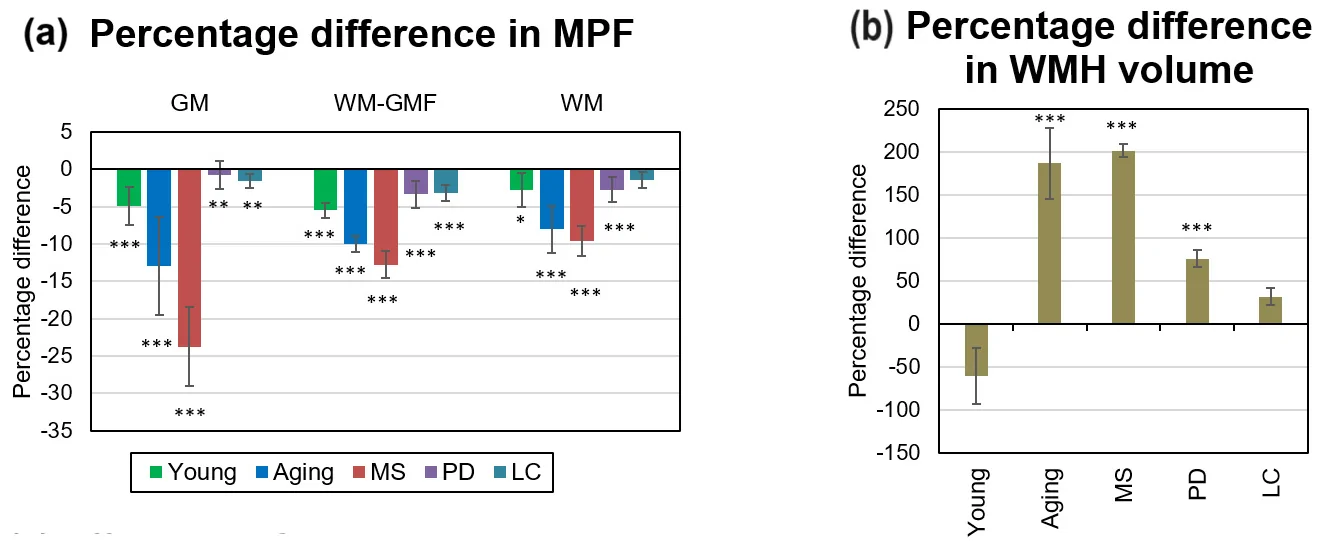

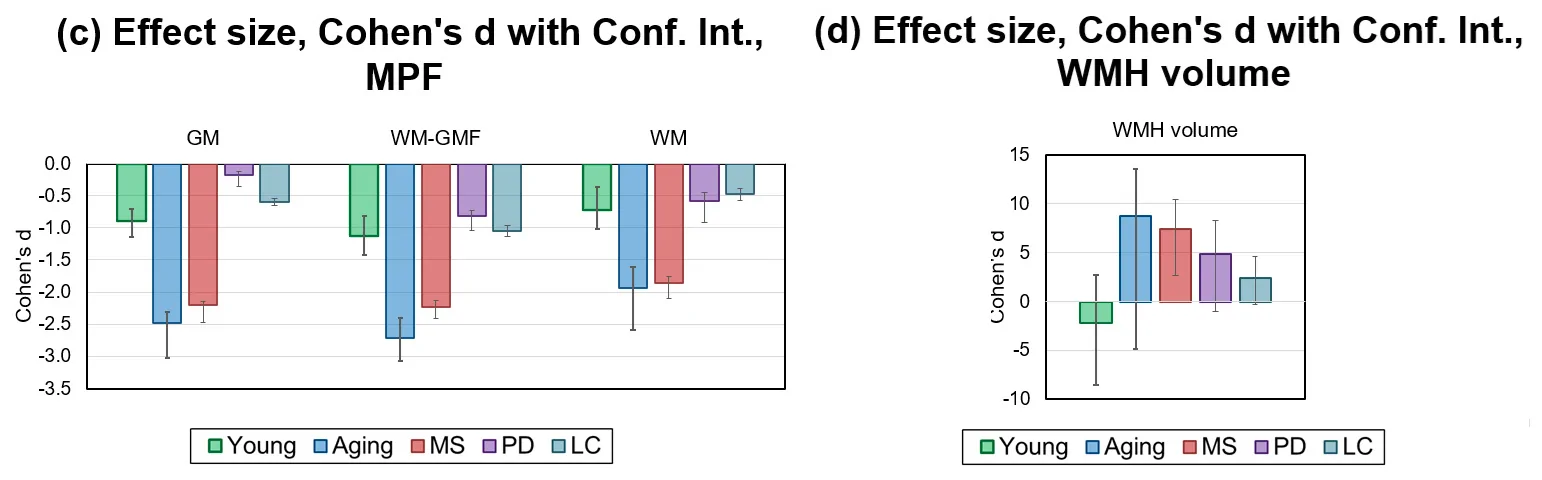
Age- and disease-related percentage differences in brain myelination relative to controls for MS, PD, and LC patients and relative to participants aged 35–44 years for the youngest (18–24 years) and oldest (75–85 years) participants. (**a**)—Percentage differences in MPF, adjusted for sex and brain atrophy (young and aging participants), and for sex, age, and brain atrophy (patient groups). (**b**)—Percentage differences in ranked WMH volumes, adjusted for sex, brain atrophy in young and aging control participants, and for age, sex, and brain atrophy for patient groups. (**c**,**d**) Effect sizes (Cohen’s d) with confidence intervals corresponding to specific group differences in MPF (**c**) and ranked WMH volumes (**d**) relative to the control groups. Significant differences relative to controls: *—*p* < 0.05, **—*p* < 0.01, ***—*p* < 0.001. Error bars correspond to CI.

Percentage differences in global and focal brain myelination adjusted by age, gender, and brain atrophy, in MS, PD, LC patients relative to controls, as well as age-related differences are summarized in Figure 6. The most prominent demyelination, including the highest MPF decrease in GM (−23.7 ± 5.3%), WM (−9.6 ± 2.0%), and WM-GM (−12.8 ± 1.8%) and WMH volume increase (201.9 ± 8% for ranked WMH, 1688.3 ± 291.7% for raw WMH) was observed in MS patients compared to controls. Lower but significant MPF decreases in GM (−0.8 ± 1.9%), WM (−2.7 ± 1.7%), WM-GM (−3.4 ± 1.9%), and an increase in WMH volume (76.3 ± 9.8% for ranked WMH, 1520.8 ± 164.5% for raw WMH) were found for PD patients compared to controls. In LC patients, small but significant MPF decrease relative to controls was found for GM (−1.6 ± 0.9%) and WM-GM (−3.2% ± 1.1%) global compartments. Age-related changes in myelin content in 75–85-year controls were second largest after those in MS compared to controls (the control group for MS patients and 35–44-year group for 75–85-year group): a significant MPF decrease in GM (−13.0 ± 6.6%), WM-GM (−9.9 ± 1.4%), WM (−8.1 ± 3.1%) compartments and an increase in WMH volume (187.3 ± 41.3% for ranked WMH, 727.3 ± 514% for raw WMH). MPF in GM (−3.9 ± 2.6%), WM (−2.8 ± 2.2%), and WM-GM (−5.5 ± 1.0%) compartments of the youngest participants also was significantly lower than in participants aged 35–44 years. Figure 6c,d compares the effect sizes characterizing differences in MPF and ranked WMH volumes across specific groups of patients (adjusted for age, sex, and brain atrophy) or age ranges (adjusted for sex and brain atrophy). The largest effect (Cohen’s d > 2) of MPF decrease was found for GM and WM-GM compartments in MS patients and the 75–85-year group relative to controls. In WM, a large effect (Cohen’s d > 0.8) was found for these groups. The youngest participants showed a large effect (Cohen’s d > 0.8) in GM and WM-GM and a small effect (0.5 > Cohen’s d > 0.2). The PD- and LC-related differences showed a small effect (0.5 > Cohen’s d > 0.2) for all compartments except for the GM in PD patients. WMH volume showed a large effect in all groups but also high variability of measurements.

## 4. Discussion

This study, for the first time, compared disease-dependent alterations in global WM and GM myelination in patients with MS, PD, and LC measured by quantitative MPF mapping. Even the initial comparison of patient groups with the control group, without taking into account differences in gender and age, revealed significant reductions in MPF in all patient groups: for all compartments in MS patients, for WM and WM-GM in PD patients, and in WM-GM for LC patients. By controlling for age, gender and brain atrophy influence, the data were analyzed in more detail, and it can be argued that the differences are due to the disease and are not related to gender, age, and brain atrophy.

As expected, the most prominent global demyelination was found in MS patients. In these patients, a significant decrease in MPF, adjusted to tissue loss and gender, was observed in all global compartments both in RRMS and SPMS patients. The largest percentage decrease in MPF was found in global GM and mixed WM-GM, and in these compartments SPMS patients showed significantly higher MPF decline compared to RRMS patients. In addition, WMH volume was significantly higher in SPMS patients compared to RRMS patients. A strong correlation between MPF and EDSS was found in GM and moderate correlations in WM-GM and WM. These results are similar to those obtained previously, where the correlation of MPF with EDSS in MS patients was strong for GM and moderate for WM [23]. However, correlations between adjusted MPF and EDSS were non-significant in contrast to raw MPF values. These results suggest that age does not have a significant effect on MS, which typically develops in middle-aged individuals.

The majority of quantitative MRI studies showed a significant demyelination in NAWM [20,21,22,23,33], but only myelin water fraction (MWF) and MPF techniques were able to detect a decrease in global GM myelination [21,23,33] in MS patients. Our findings are in accordance with previous studies [23,33], which investigated demyelination in MS patients using MPF mapping. The first study [23] showed a greater MPF decrease and its stronger correlation with clinical scores in GM compared to WM. The second study [33] showed a significant MPF decrease in deep GM structures and its correlation with clinical scores in MS patients. Rahmanzadeh et al. [21] found a decreased MWF both in WM and GM compared with healthy controls, and in SPMS patients compared to RRMS patients. Histopathological studies found that the normal-appearing GM of MS patients is characterized by myelin disturbances with the loss of particular neuronal populations, synaptic demise, and microglial alterations compared with the GM of non-neurological controls [22]. Our results confirmed the important role of GM demyelination in MS pathology.

In PD patients, a smaller (compared to MS patients) but significant reduction in MPF, independent of age influence, was found in all compartments compared to controls. WMH volume in PD patients was significantly higher in comparison with controls. Furthermore, significant moderate correlations were found between MPF and PD stage that makes MPF a valuable prognostic marker for PD. These correlations persist for age-adjusted MPF.

A number of studies investigated changes in WMH volume and normal-appearing WM and GM in PD [5,7,16,24,25,60,61,62]. Most studies found an increased WMH volume in PD patients compared to age-matched controls [16,17,60]. In DTI studies, alterations both in WM and GM were found compared to age-matched controls [25,61,62] but one study did not show differences [60]. Given iron deposition in the subcortical nuclei in older age and its impact on DTI [63], such measurements in GM should be considered with some caution. For the MPF mapping method, independence on iron deposition in the deep GM was demonstrated in the previous study [33]. Unlike some previous studies [25,61,62], we did not find significant differences in NAWM in PD patients compared to controls. It is possible that the small sample size limited our ability to detect these differences. However, the differences in study results may also be explained by the different specificity of MPF and DTI to myelin. Further studies involving a larger number of PD patients and a combination of the study of demyelination with other proven diagnostic markers in PET imaging [64] will help clarify this issue.

LC patients showed the least MPF reduction compared to MS and PD patients, but these differences were significant in the GM and WM-GM compartments. Significant MPF reductions (primarily in WM-GM) were found for depression, insomnia, chronic headache, asthenia, cognitive impairment, and hyposmia as complications of COVID-19. Among all subsamples of patients with specific post-COVID complications, the greatest MPF reduction was found in LC patients with depression and insomnia. In these patients, a significant MPF reduction was observed not only compared to the control group but also compared to other LC patients without insomnia or depression. A significant increase in WMH volume compared to controls and other LC patients was found only for LC patients with cognitive impairment. Our results are consistent with our previous studies [27,65,66] that found significant MPF reduction in GM and WM brain structures in patients with post-COVID depression, insomnia, and cognitive impairments. Our previous results [66] also demonstrated MPF reduction within commissural and projection WM tracts in patients with LC and insomnia, whereas LC depression has a less pronounced impact on pure WM but selectively affects the basal ganglia and subcortical pathways (corresponding to WM-GM global compartment in the present study).

Changes in WM as a consequence of COVID-19 were found in many DTI studies [67,68,69,70,71,72,73,74] but few studies considered WM changes in relation to the specific symptoms of LC patients. We found several studies (including our [27,66]) in LC patients that examined WM changes in relation to depression and insomnia [69,75,76]. Cui et al. [69] found decreased FA in WM tracts in patients with severe depression compared to patients with mild or moderate depression after one COVID-19 infection. Insomnia- and depression-related psychological scores were negatively correlated with the FA in these WM tracts. The study by Benedetti et al. [75] showed negative associations between AD in several WM tracts and self-rated depression. Qin et al. [76] found lower FA and higher RD and AD in acute COVID-19 patients with sleep disorders compared to healthy controls. These WM abnormalities correlated with insomnia scores. Three months after recovery, insomnia-related scores slightly improved, depression- and anxiety-related scores remained unchanged, and DTI measures changed significantly only in two brain structures. Unfortunately, healthy participants were not included in two [69,75] of the three above studies; therefore, the lack of control groups does not allow us to compare these studies with our results. The problem lies not only in the insufficient number of studies, but also in the lack of direct comparisons between LC patients diagnosed with insomnia and depression, and patients in whom these disorders are not consequences of COVID-19. Such studies would help to better understand the specificity in the pathophysiology of depression and insomnia caused by viral infections.

We did not find significant correlations between global MPF values and the severity of the acute phase of COVID-19-caused LC. Some previous DTI studies have demonstrated relationships between WM changes and the severity of the acute phase of COVID-19 [70,71]. However, our recent studies have shown that WM changes in LC are region-specific for particular LC complications, such as insomnia and depression, and are less dependent on the severity of the acute phase of COVID-19 [27,66].

The present study refined the patterns of age-related differences in MPF obtained in previous studies [31,32] on a larger sample of healthy volunteers. Overall, the previously made estimates of the peak age of myelination in WM-GM and WM were similar: in the previously conducted study these estimates were 46.9 and 43.1 years, in the present study 46.5 and 42.2 years, respectively. In both studies, a later peak age for WM-GM and WM compartments was observed in females, but the present study found that these differences are smaller (1.4 and 0.3 years) than those obtained previously (4.6 and 2.1 years). The present study also determined the peak age of myelination for GM as 45.7 years in men and 45.1 years in women. Compared with the group with maximal myelination (35–44 years), we found significantly lower MPF values in all compartments for participants in both the oldest (75–85 years) and youngest (18–24 years) age groups. The percentage reduction in MPF associated with normal aging was even greater than age-adjusted MPF in PD patients as compared with controls.

Despite numerous studies investigating age-related changes in myelination [31,55,77,78,79,80,81,82,83,84], they are often limited to the linear model [77,81,82] but a more appropriate quadratic (inverted-U) model was also used for wider age ranges [84,85,86,87]. Within a linear model, positive correlations were found in childhood and adolescence [55] while negative correlations were found at ages over 60 years [79,81]. Westley et al. [85] within a quadratic model estimated WM peak age of myelination at 29.1 years based on FA, 35.7 years based on MD, and 31.1 years based on RD. Based on DTI indices, peak age of WM myelination was estimated by Kochunov et al. [87] as 23–39 years, and by Lebel et al. [83] as 21–44 years old. Arshad et al. [86] studied age-related changes in myelination using DTI and WMF data within linear and quadratic models. It was found that DTI parameters, FA and RD, correlate with age only within the linear model, while more myelin-specific MWF correlates with age within the quadratic model. In this study, the peak age of myelination for several WM tracts was estimated around the fourth-sixth decade of life. In the study by Kieley et al. [88], the peak age of myelination is estimated at 43.76 years, which is close to our estimates. In the same study, all DTI parameters (FA, MD, RD, and AxD) yield lower estimates at 30–35 years. Our estimates for the peak age of global myelination (42.2 years) are close to those obtained using the MWF method, which is considered more accurate for quantifying myelin than DTI. We did not find any studies in the literature assessing the peak age of myelination in GM.

Brain tissue atrophy commonly coincides with myelin loss in normal brain aging [89] and various neurological conditions including MS [90] and primary neurodegenerative disorders, such as Alzheimer’s disease and PD [5]. Recent studies also suggest associations between LC and neurodegeneration [91]. While this study is focused on myelin, a possible effect of brain tissue loss on MPF measurements needs to be considered. Atrophy, particularly in GM, may increase the proportion of voxels affected by partial volume averaging with CSF and cause a reduction in observed MPF values [92]. To avoid a confounding effect of atrophy, we applied a rigorous methodology involving both a specialized brain tissue segmentation scheme and statistical control for this potential confounder in all relevant analyses. Specifically, the use of four-class segmentation was shown to minimize the partial volume effect on the MPF measurements in pure WM and GM [23]. The use of ANCOVA with tissue volumes as covariates validates the significance of the observed MPF differences in isolation from the confounding effect of tissue loss, which indeed appeared significant in the studied diseases (MS, PD, LC) and normal aging. As such, our results provide compelling evidence of myelin alterations and establish a reliable quantitative scale of demyelination in the above conditions.

Our results highlight the capability of fast MPF mapping to detect very subtle GM differences in brain myelination that correlate with clinical data. Since many studies consider myelin loss as the basis of cognitive decline [93,94,95,96,97,98], it would be of great interest to investigate associations of demyelination with performance on cognitive tests. Future studies will define the contribution of demyelination of specific brain structures to age- and disease-related decline of cognitive abilities. Our results indicate that MPF decrease, which strongly correlates with demyelination of normal-appearing WM and GM, is observed not only in traditionally studied primary demyelinating diseases such as MS, but also in diseases where demyelination is not as obvious and is rarely studied (PD and LC). Future research could focus on studying the role of alterations of normal-appearing WM and GM in other diseases, including cerebrovascular diseases, dementia and Alzheimer’s disease, major depressive disorder, autism, and others. Intensively developing artificial intelligence algorithms, particularly for accurate segmentation of lesions and their differentiation [99,100,101,102], could play an important role in such studies.

## 5. Conclusions

This is the first study that compared disease-dependent differences in global WM and GM myelination in patients with MS, PD, LC, and in normal aging using quantitative MPF mapping. The inclusion of age and gender covariates allowed us to separate the contributions of specific diseases to the decrease in myelin content from the contributions of gender and age. These differences in MPF values are also independent of brain atrophy, which is observed in patients with MS, PD, and in elderly individuals.

Significant WMH volume increase and MPF decrease in global WM, WM-GM, and GM were found in both normal aging and MS, PD, and LC patients. The greatest MPF decrease was observed in MS patients. Significant MPF reduction was found in all global compartments in both RRMS and SPMS patients. The differences between RRMS and SPMS patients were significant for global GM and WM-GM.

Of particular clinical interest are significant correlations between global MPF values and clinical data in MS and PD patients. This relationship was demonstrated for the first time in patients with PD. The strongest correlation was found between MPF in GM and EDSS in MS patients, emphasizing the importance of GM demyelination in MS.

The study also, for the first time, showed significant global differences in LC patients. Among LC patients, the greatest MPF reduction was found in patients with depression and insomnia as COVID-19 complications.

The present study refined the patterns of age-related differences in myelination obtained in previous studies [31,32] on a larger sample of healthy controls. The peak age of myelination was estimated as 42.2 years for WM, 46.5 years for WM-GM, and 45.4 years for GM.

Both MS, PD, and LC patients and the oldest (75–85 years) controls revealed the greatest MPF decrease in global GM. These results highlight the importance of GM demyelination in disease pathology and normal aging. MPF mapping showed high sensitivity to age-related and disease-related differences in brain myelination, suggesting the feasibility of clinical applications of this method.

## 6. Study Limitations

Our study has an imbalance in sample sizes between disease-specific groups. In particular, the subgroups of healthy elderly participants, the group of patients with PD and the subgroups with specific LC complications have small sample sizes, which reduces the statistical power to detect significant differences. All LC patients were newly diagnosed with neurological and psychiatric conditions after COVID-19. Therefore, it is unclear whether the observed differences in MPF are related to post-COVID complications in general or only to neuropsychiatric complications. Also, we cannot fully guarantee that the occurrence of these conditions was not influenced by sociodemographic factors associated with the pandemic.

## Figures and Tables

**Table 1:** The demographic and clinical characteristics of the patients and controls.

Parameter	MS Patients	PD Patients	LC Patients	Control Group
Sample size	42	16	75	63
Male (%)/Female (%)	9(21)/33(79) $	5(31)/11(69)	25(17)/50(83)	28(44)/35(56)
Age, years ± SD	39.0 ± 9.5	69.7 ± 9.1 ***	40.8 ± 11.7	41.7 ± 18.3
Age, median (min–max)	39(25–67) ###	70(51–85) ##	42(19–61) ###	40(18–85)
Disease duration, years ± SD	9.0 ± 5.7	9.8 ± 3.9	1.8 ± 0.8 ^2^	-
Disease severity, parameters (%)	RR/SP (71/29)	Stage ^1^ 1/2/3/4 (6/19/50/25)	mild/moderate/severe/critical ^3^ (75/11/11/3)	-

Data are presented as mean ± SD. Significant differences relative to the control group: $—*p* < 0.05, Fisher exact test for gender ratio; ***—*p* < 0.001, Fisher LSD test for mean age difference; ##—*p* < 0.01, ###—*p* < 0.001, Levene’s test of homogeneity of variance in age range. ^1^—stage of PD according to the Hoehn and Yahr scale. ^2^—time after recovery from the first COVID-19 episode. ^3^—severity of COVID-19-caused LC.

**Table 2:** The comparison of patient groups with the control group.

Sample	MPF in WM	MPF in WM-GM	MPF in GM	WMH Volume
Control group	13.71 ± 0.12	10.43 ± 0.09	7.56 ± 0.08	0.96 ± 0.81
MS patients	12.75 ± 0.25 ***	9.20 ± 0.10 ***	6.03 ± 0.33 ***	10.87 ± 3.11 ***
PD patients	13.19 ± 0.37 *	10.03 ± 0.26 **	7.24 ± 0.20	12.35 ± 7.18 ***
LC patients	13.68 ± 0.10	10.19 ± 0.07 *	7.47 ± 0.05	0.96 ± 0.36

Data are presented as mean ± CI. Significant differences relative to the control group: *—*p* < 0.05, **—*p* < 0.01, ***—*p* < 0.001 (Welch test with FDR correction).

## Data Availability

The data presented in this study are available on request from the corresponding author.

## Supplementary Materials

The following supporting information can be downloaded at: https://www.mdpi.com/article/10.3390/biomedicines14081691/s1, Table S1: Significance (*p*-values) of differences in demographic characteristics between groups of patients and control groups; Figure S1: Raincloud plots presented group differences in age according to Levene’s test of homogeneity of variance; Text S1: Formal Mathematical Specification of the General Linear Models; Supplementary Table S2: Significance of intergroup differences (*p*-values) of Bonferroni (Bonf) and Welch tests; Table S3: Significance of age-, sex-, and brain atrophy-adjusted intergroup differences in MPF and ranked WHM volumes; Table S4: Significance of covariates (gender, age squared, volume of the WM, GM, and WM-GM compartments) for GLM presented in Table S3; Table S5. The parameters of the quadratic models of the dependence of MPF on age; Table S6. The results of a Leave-One-Out analysis for the quadratic dependence of MPF on age.
