# Supplementary material for: MRI-Based Quantitative Assessment of Normal-Appearing White and Gray Matter Demyelination in Multiple Sclerosis, Parkinson’s Disease, Long COVID, and Normal Aging
Source: Biomedicines. 2026 Jul 28;14(8):1691. doi: 10.3390/biomedicines14081691 (PMC13509534; doi:10.3390/biomedicines14081691)
Supplement: Supplementary file 1 [file biomedicines-14-01691-s001.zip › biomedicines-4364696-supplementary.pdf]

Supplementary Table S1. Significance (p-values) of differences in demographic characteristics between groups of patients and control groups

Disease	Table	Comparison group	Mean age (Fisher LSD test)	Age range (Levene's test)	Male/Female (Fisher exact test)
MS	1	Total sample of healthy participants	0.134	0.000	0.018
	2	MS control group	0.216	0.318	0.177
PD	1	Total sample of healthy participants	0.0000	0.004	0.291
	2	PD control group	0.212	0.399	0.549
LC	1	Total sample of healthy participants	0.617	0.000	0.165
	2	LC control group	0.780	0.722	0.283

Supplementary Figure S1. Raincloud plots presented group differences in age according to Levene's test of homogeneity of variance. (a) Significant differences between the general control group (Control) and MS, PD, and LC patients. (b-d) Non-significant differences of age-matched control groups with MS (b), PD (c), and LC (d) patients.

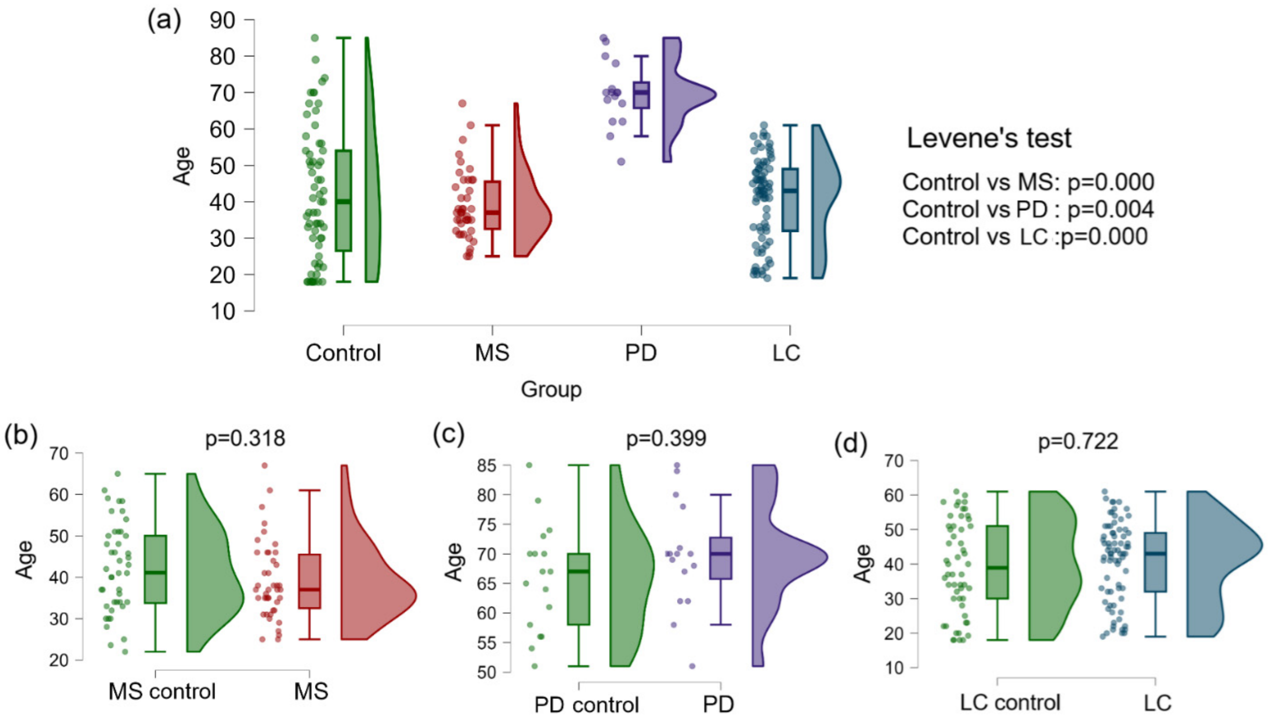

Supplementary Table S2. Significance of intergroup differences (p-values) of Bonferroni (Bonf) and Welch tests.

Sample	MPF in WM		MPF in WM-GM		MPF in GM		WMH volume	
	Bonf test	Welch test	Bonf test	Welch test	Bonf test	Welch test	Bonf test	Welch test
MS patients	0.000	0.000	0.000	0.000	0.000	0.000	0.000	0.000
PD patients	0.007	0.010	0.009	0.008	0.032	0.004	0.000	0.004
LC patients	1.00	0.870	0.010	0.003	1.00	0.154	1.00	0.769

Text S1. Formal Mathematical Specification of the General Linear Models

1. **Preliminary Model.**

A standard one-way ANOVA framework utilized for primary group comparisons without covariate adjustments followed by post-hoc Bonferroni tests. Welch's ANOVA was additionally applied in cases of unequal variances.

2. **Main Model**

To investigate the determinants of brain tissue characteristics, a unified General Linear Model (GLM) approach was applied separately to each quantitative parameter: white matter hyperintensities (WMH), macromolecular proton fraction (MPF) in WM, WM-GM, and GM. For all parameters, the model evaluated the main effects of patient group, sex, their interaction, compartment volume (adjusting for brain atrophy), and age effects via a parameter-specific function f(age).

For WMH parameter, f(age) represented the standard linear age variable:

$$f(age_i) = age_i \quad (1)$$

For MPF parameter, f(age) represented a compartment-specific composite quadratic age covariate derived from a preliminary normative regression calculated within healthy controls:

$$f(age_i) = c + b \times age_i + a \times age_i^2 \quad (2),$$

where *c* is the constant intercept, *b* and *a* are the linear and quadratic regression coefficients, specific for each compartment (WM, WM-GM, GM), estimated from the healthy control sample.

Preliminary analyses confirmed the homogeneity of slopes ($p > 0.05$) for all factor-by-covariate interactions). Residuals for all MPF models followed a normal distribution (Shapiro-Wilk test, $p > 0.05$). However, residuals for raw WMH volumes significantly deviated from normality ($p < 0.05$) due to high age-dependent variance. To meet GLM assumptions, all volumetric WMH data were log-transformed (log(WMH + 1)) prior to analysis, which successfully restored residual normality ($p > 0.05$).

Preliminary analyses confirmed the homogeneity of slopes ($p > 0.05$) for all factor-by-covariate interactions. Residuals for all MPF models followed a normal distribution

(Shapiro-Wilk test, $p > 0.05$). However, residuals for raw WMH volumes significantly deviated from normality ($p < 0.05$) due to high age-dependent variance. To meet GLM assumptions, a non-parametric Rank ANCOVA via the Conover–Iman procedure was applied, where all WMH data were transformed into continuous ranks across the entire sample prior to analysis, which successfully restored residual normality ($p > 0.05$)

The final statistical model for the i-th participant is formalized as follows:

$$Y_i = \beta_0 + \sum\beta_{\{group\}} \cdot Group_{\{ij\}} + \sum\beta_{\{sex\}} \cdot Sex_{\{ik\}} + \sum\beta_{\{inter\}} \cdot (Group \times Sex)_{\{ijk\}} + \beta_1 \cdot Vol_i + \beta_2 \cdot f(Age_i) + \epsilon_i \quad (4)$$

Where:

- Y_i - the analyzed dependent variable (raw MPF or globally ranked WMH volumes) for i-th participant,
- β_0 - the model intercept.
- $Group_{ij}$ - dummy-coded variables representing study groups,
- Sex_{ik} - the binary variable representing sex of participants,
- $(Group \times Sex)_{ijk}$ - the interaction between group and sex,
- Vol_i – the compartment-specific linear covariate for WM, WM-GM or GM volume for MPF parameter; WM volume for WMH parameter,
- $f(Age_i)$ - the parameter-specific age covariate, compartment-specific quadratic for MPF (2) and linear for WMH (1),
- $\beta_{\{group\}}$, $\beta_{\{sex\}}$, $\beta_{\{inter\}}$, β_1, β_2 - the estimated regression coefficients.
- ε - the random error term.

Pairwise intergroup differences were evaluated using post-hoc Bonferroni test.

3. Specific Model to assess symptom overlap in LC patients

To investigate the overlap of insomnia and depression symptoms within the LC cohort and control for clinical data, an expanded model was constructed for LC patient subgroups (insomnia, depression, insomnia+depression, LC without insomnia/depression). The expanded model for the i-th participant is formalized as:

$$Y_i = \beta_0 + \sum\beta_{\{subgroup\}} \cdot Subgroup_{\{ij\}} + \sum\beta_{\{sex\}} \cdot Sex_{\{ik\}} + \beta_1 \cdot Vol_i + \beta_2 \cdot f(age_i) + \beta_3 \cdot Severity_i + \beta_4 \cdot Time_i + \epsilon_i \ (5),$$

Where the model includes the following additional clinical predictors:

- $Subgroup_{ij}$ = dummy-coded categorical factor (LC insomnia without depression, LC combined insomnia-depression, LC depression without insomnia, LC without insomnia/depression, and healthy controls),
- $Severity_i$ = a categorical covariate representing the acute severity stage (mild, moderate, severe, critical) of the COVID-19 infection.
- $Time_i$ = a continuous linear covariate representing the time from acute COVID-19 recovery in months.
- Medication status was not included in the model as all participants were treatment-naive at the time of enrollment.

Supplementary Table S3. Significance of age-, sex-, and brain atrophy-adjusted intergroup differences in MPF and ranked WHM volumes. The results (p-values) of Bonferroni tests for multiple comparisons.

Study	Fig.	Comparison	MPF in WM	MPF in WM-GM	MPF in GM	WMH volume*
Aging	2, b, c<br>6, a, b	25-34-year group vs 18-24-year group	0.211	0.002	0.007	1.00
		35-44-year group vs 18-24-year group	0.007	0.000	0.0008	0.782
		45-54-year group vs 18-24-year group	0.325	0.001	0.000	0.103
		55-64-year group vs 18-24-year group	0.712	0.033	0.019	0.002
		65-74-year group vs 18-24-year group	0.922	0.041	0.031	0.003
		75-85-year group vs 18-24-year group	0.026	0.311	0.117	0.000
		75-85-year group vs 25-34-year group	0.003	0.005	0.001	0.000
		75-85-year group vs 35-44-year group	0.000	0.000	0.000	0.000
		75-85-year group vs 45-54-year group	0.007	0.003	0.000	0.000
		75-85-year group vs 55-64-year group	0.014	0.018	0.005	0.001
		75-85-year group vs 65-74-year group	0.067	0.019	0.008	0.136
PD	4, b, c<br>6, a, b	PD patients vs controls	0.905	0.000	0.001	0.000
MS	6, a, b	MS patients vs controls	0.000	0.0000	0.000	0.0000
	3, b, c	RR patients vs controls	0.000	0.0000	0.0000	0.0000
		SP patients vs controls	0.000	0.0000	0.0000	0.0000
		RR patients vs SP patients	0.575	0.002	0.0000	0.446
LC	6, a, b	LC patients vs controls	0.022	0.000	0.0000	0.964
	5, c-f	LC patients with depression vs controls	0.06	0.000	0.000	0.507
		LC patients: with vs without depression	1.00	0.002	0.007	0.052
		LC patients with chronic headache vs controls	0.23	0.003	0.27	0.087
		LC patients: with vs without headache	1.00	1.00	1.00	0.104
		LC patients with hyposmia vs controls	1.00	0.337	1.00	0.518
		LC patients: with vs without hyposmia	0.65	0.136	0.153	0.742
		LC patients with asthenia vs controls	1.00	0.000	0.065	0.053
		LC patients: with vs without asthenia	0.358	1.00	1.00	1.00

		LC patients with cognitive impairment vs controls	0.031	0.0000	0.344	0.000
		LC patients: with vs without cognitive impairment	0.069	0.001	0.630	0.000
		LC patients with insomnia vs controls	0.001	0.000	0.003	0.009
		LC patients: with vs without insomnia	0.017	1.00	0.017	0.066

Supplementary Table S4. Significance of covariates (gender, age squared, volume of the WM, GM, and WM-GM compartments) for GLM presented in table S3. *P*-values for covariates are presented as: gender/compartment volume/f(age)

Study	Categorical factor	Levels	MPF in WM (sex/volume/f(age))	MPF in WM-GM (sex/volume/f(age))	MPF in GM (sex/volume/f(age))	WMH volume (sex/volume/age)
Aging	Age range		0.03/0.01/-	0.330/0.148/-	0.020/0.008/-	0.0000
PD	PD	Patients, controls	0.710/0.091/0.000	0.053/0.001/0.000	0.727/0.03/0.000	0.96/0.56/0.000
MS	MS	Patients, controls	0.225/0.062/0.030	0.03/0.008/0.000	0.910/0. 104/0.032	0.86/0.09/0.009
	MS diagnosis	RRMS, SPMS, controls	0.793/0.127/0.000	0.142/0.110/0.000	0.315/0.127/0.001	0.44/0.22/0.000
LC	LC	Patients, controls	0.01/0.06/0.000	0.000/0.73/0.000	0.67/0.000/0.000	0.27/0.65/0.000
	Depression	Depression, controls	0.26/0.07/0.000	0.011/0.70/0.000	0.26/0.07/0.000	0.44/0.81/0.000
	Headache	Headache, controls	0.13/0.07/0.000	0.01/0.72/0.000	0.84/0.000/0.000	0.48/0.72/0.000
	Hyposmia	Hyposmia, controls	0.016/0.0.34/0.000	0.000/0.46/0.000	0.74/0.000/0.000	0.31/0.61/0.000
	Asthenia	Asthenia, controls	0.045/0.036/0.000	0.000/0.87/0.000	0.95/0.000/0.000	0.41/0.67/0.000
	Cognitive impairment	Cognitive impairment, controls	0.043/0.034/0.000	0.000/0.73/0.000	0.92/0.000/0.000	0.37/0.84/0.000
	Insomnia	Insomnia, controls	0.037/0.032/0.000	0.000/0.73/0.000	0.84/0.000/0.000	0.36/0.68/0.000

Supplementary Table S5. The parameters of the quadratic models of the dependence of MPF on age. The coefficients of quadratic equation ($A*x^2+B*x+C$) are presented with low and upper limits (confidence intervals, CI)

Compartment	Sample	n	F	R	R^2	p	A (0.95% CI), p	B (0.95% CI), p	C (0.95% CI), p
WM	men	28	15.2	0.74	0.55	0.000	-0.0009 (-0.001, -0.0005), 0.000	0.071 (0.039, 0.106), 0.000	12.71 (11.99, 13.35), 0.000
	women	35	10.8	0.63	0.40	0.000	-0.0007 (-0.001, -0.0004), 0.000	0.061 (0.028, 0.095), 0.000	12.50 (11.77, 13.22), 0.000
	pooled	63	20.3	0.63	0.40	0.000	-0.0008 (-0.001, -0.0005), 0.000	0.069 (0.039, 0.090), 0.000	12.62 (12.1, 13.2), 0.000
WM-GM	men	28	14.1	0.73	0.53	0.000	-0.001 (-0.001, -0.0005), 0.000	0.089 (0.058, 0.121), 0.000	8.82 (8.19, 9.46), 0.000
	women	35	14.9	0.74	0.55	0.000	-0.0008 (-0.001, -0.0005), 0.0000	0.070 (0.043, 0.097), 0.0000	8.98 (8.40, 9.56), 0.0000
	pooled	63	26.0	0.71	0.50	0.000	-0.0008 (-0.001, -0.0006), 0.000	0.077 (0.055, 0.098), 0.0000	8.94 (8.49, 9.39), 0.0000
GM	men	28	14.5	0.79	0.63	0.000	-0.0007 (-0.0009, -0.0005), 0.000	0.064 (0.043, 0.085), 0.000	6.38 (5.95, 6.80), 0.000
	women	35	16.4	0.74	0.55	0.000	-0.0006 (-0.0008, -0.0004), 0.000	0.056 (0.035, 0.077), 0.000	6.51 (6.06, 6.96), 0.000
	pooled	63	31.6	0.76	0.58	0.000	-0.0006 (-0.0008, -0.0005), 0.000	0.057 (0.045, 0.073), 0.000	6.50 (6.16, 6.76), 0.000

Supplementary Table S6. The results of a Leave-One-Out analysis for the quadratic dependence of MPF on age. The omitted extreme data point corresponded to the maximal absolute standardized residual.

Compartment	Sample	N (f/o*)	Model, R (f/o), p (o)	Residuals normality, p (f/o)	A (f/o), p (o)	B (f/o), p (o)	C (f/o), p (o)
WM	men	28/27	0.74/0.76, 0.000	0.67/0.43	-0.00085/-0.00076, 0.000	0.071/0.076, 0.000	12.71/12.97, 0.000
	women	35/34	0.63/0.65, 0.000	0.26/0.35	-0.00071/-0.00067, 0.000	0.061 (0.026, 0.095), 0.000	12.50/12.61, 0.000
	pooled	63/62	0.63/0.63. 0.000	0.80/0.65	-0.00077/-0.00074, 0.000	0.069/0.062, 0.000	12.62/12.69, 0.000
WM-GM	men	28/27	0.73/0.78, 0.000	0.49/0.98	-0.00096/0.0011, 0.000	0.089/0.080, 0.000	8.82/8.97, 0.000
	women	35/34	0.74/0.74, 0.000	0.32/0.29	-0.00075/0.00086, 0.000	0.070/0.064, 0.000	8.98/9.07, 0.000
	pooled	63/62	0.71/0.71, 0.000	0.31/0.88	-0.00083/-0.00092, 0.000	0.077/0.074, 0.000	8.94/9.00, 0.000
GM	men	28/27	0.74/0.78	0.41/0.41	-0.00070/-0.00071, 0.000	0.064/0.064, 0.000	6.37/6.38, 0.000
	women	35/34	0.72/0.79, 0.000	0.15/0.46	-0.00062/-0.00062, 0.000	0.056/0.54, 0.000	6.54/6.56, 0.000
	pooled	63/62	0.76/0.78, 0.000	0.22/0.25	-0.00065/-0.00065, 0.000	0.057/0.065, 0.000	6.50/6.48, 0.000

* - f – full sample, o – sample with omitted a single extreme data point
